# TLC-Bioautography-Guided Isolation and Assessment of Antibacterial Compounds from Manuka (*Leptospermum scoparium*) Leaf and Branch Extracts

**DOI:** 10.3390/molecules29030717

**Published:** 2024-02-04

**Authors:** Wenliang Xu, Danxia Shi, Kuanmin Chen, David G. Popovich

**Affiliations:** 1 School of Food and Advanced Technology, Massey University, Palmerston North 4410, New Zealand; w.xu1@massey.ac.nz (W.X.); sdanqiu@outlook.com (D.S.); k.chen@massey.ac.nz (K.C.); 2 School of Science, Engineering & Technology, RMIT, Ho Chi Minh City 800010, Vietnam

**Keywords:** Manuka, *Leptospermum scoparium*, TLC-bioautography, LC-MS, compound discoverer, grandiflorone, NMR, antibacterial activity

## Abstract

A rapid procedure for the targeted isolation of antibacterial compounds from Manuka (*Leptospermum scoparium*) leaf and branch extracts was described in this paper. Antibacterial compounds from three different Manuka samples collected from New Zealand and China were compared. The active compounds were targeted by TLC-bioautography against *S. aureus* and were identified by HR-ESI-MS, and -MS/MS analysis in conjunction with Compound Discoverer 3.3. The major antibacterial component, grandiflorone, was identified, along with 20 β-triketones, flavonoids, and phloroglucinol derivatives. To verify the software identification, grandiflorone underwent purification via column chromatography, and its structure was elucidated through NMR analysis, ultimately confirming its identity as grandiflorone. This study successfully demonstrated that the leaves and branches remaining after Manuka essential oil distillation serve as excellent source for extracting grandiflorone. Additionally, we proposed an improved TLC-bioautography protocol for evaluating the antibacterial efficacy on solid surfaces, which is suitable for both *S. aureus* and *E. coli*. The minimum effective dose (MED) of grandiflorone was observed to be 0.29–0.59 μg/cm^2^ against *S. aureus* and 2.34–4.68 μg/cm^2^ against *E. coli*, respectively. Furthermore, the synthetic plant growth retardant, paclobutrazol, was isolated from the samples obtained in China. It is hypothesized that this compound may disrupt the synthesis pathway of triketones, consequently diminishing the antibacterial efficacy of Chinese Manuka extract in comparison to that of New Zealand.

## 1. Introduction

*Leptospermum scoparium* J.R. et G. Forst, also known as Manuka, is a member of the family *Myrtaceae,* of which all species are woody and produce essential oils [1]. The Manuka tree was originally growing throughout New Zealand [2] and in eastern Australia [3], and was used as herbal medicine by Māori people for hundreds of years [1]. This plant is well known as the source of essential oil and the nectar source for Manuka honey [4,5]. High concentrations of volatile β-triketones are characteristic compounds and the main antibacterial component in its essential oils [6,7]. The essential oil from the East Cape region of New Zealand’s North Island is the most well-known [8,9].

Β-triketone, and specifically grandiflorone [7], is both antimicrobial and herbicidal [10,11]. It only occurs in essential oils and is difficult to extract by steam distillation due to its low volatility [6,8,9,12]. The increasing interest in the potential medicinal and cosmetic uses of essential oil has led to the emergence of more oil industries [13,14]. Concurrently, the production of Manuka leaf and branch waste is also on the rise, with mulching being the only known method of utilizing this waste [15]. However, there is currently a lack of research reports on the chemical composition of the by-products obtained from essential oil industry. We speculated that the by-product left over after Manuka essential oil production (steam-distilled Manuka leaves and branches) could contain valuable bioactive molecules, especially grandiflorone. Manuka is also widely cultured in southern and eastern China with a trade name ‘Song Hongmei’ or ‘Australian Tea Tree’ [16] as an ornamental shrub. At present, there is a dearth of comparative research examining the major antibacterial components present in Manuka plants cultivated in China and New Zealand.

The objective of this study was to rapidly identify and isolate antibacterial components in Manuka leaves and branches that were surplus after essential oil extraction. At the same time, a comparative analysis of the antibacterial compounds in steam-distilled, untreated New Zealand Manuka and untreated Chinese Manuka was conducted. This study also provided an improved direct TLC-bioautography method for the osmotically vulnerable bacteria *E. coli* and established a TLC-bioautography-based minimum effective dose (MED) determination method to assess the antibacterial ability of tested compounds on the plate surface.

## 2. Results and Discussion

### 2.1. Analysis of β-Triketones

**Figure 1:**
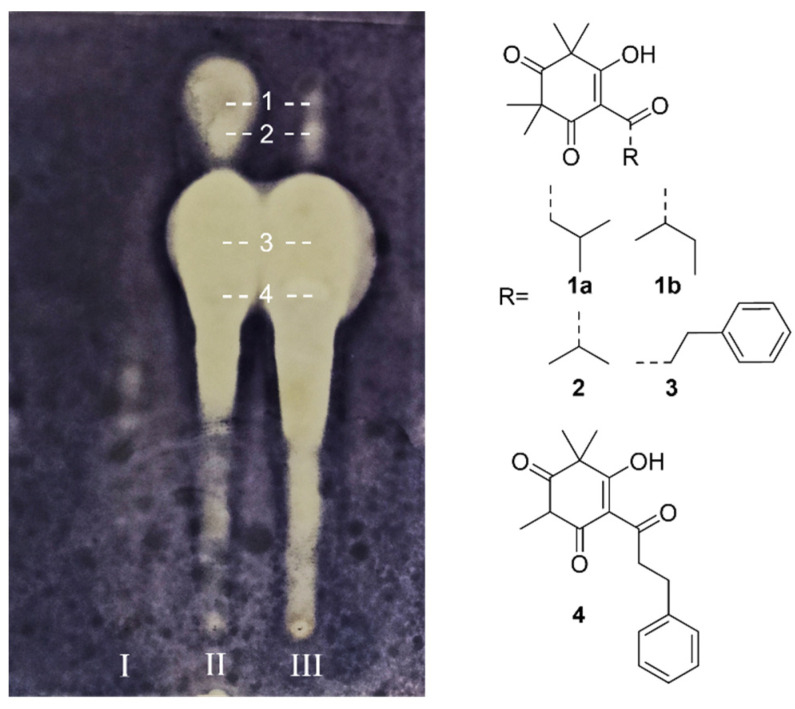
Bioautography assay of Manuka hexane extract (1 mg/mL, 3 μL spotting) against *S. aureus* at 37 °C, 16 h. Sample I: untreated CN Manuka; Sample II: untreated NZ Manuka; Sample III: steam-distilled NZ Manuka. Compounds **1**–**4** are β-triketones detected from the corresponding inhibition zones, including: a pair of isomers leptospermone (**1a**) and isoleptospermone (**1b**); flavesone (**2**); grandiflorone (**3**); myrigalone A (**4**).

This experiment simultaneously analyzed and compared the antibacterial compounds in the hexane extracts of three different Manuka samples. The hexane extract of untreated Chinese (CN) Manuka (Figure 1-I), untreated New Zealand (NZ) Manuka (Figure 1-II) and steam-distilled New Zealand (NZ) Manuka (Figure 1-III) exhibited different antibacterial effects. Under the same concentration (1 mg/mL) and the same spotting volume (3 μL), no clear inhibition zone can be observed from the CN untreated Manuka sample. Therefore, we analyzed the corresponding compounds in the inhibition zone displayed by two NZ Manuka samples. The identical TLC separation was repeated five times, and the compounds in the center of each inhibitory zone were collected based on the *R_f_* value. Through LC-MS and Compound Discoverer analysis, we discovered that there are four inhibition zones that were formed by a single main compound; the other inhibition zones were caused by complex mixtures. The position collected and the structure of major active compounds (**1**–**4**) are displayed in Figure 1.

The four major antibacterial compounds are leptospermone (**1a**) and isoleptospermone (**1b**), a pair of isomers that were unable to be separated by the current TLC and HPLC method; flavesone (**2**), grandiflorone (**3**), and myrigalone A (**4**). These compounds belong to β-triketones and are the main antibacterial ingredients in Manuka [12,17]. Among them, **1a**, **1b** and **2** are also characteristic components in Manuka essential oils [6,7].

**Figure 2:**
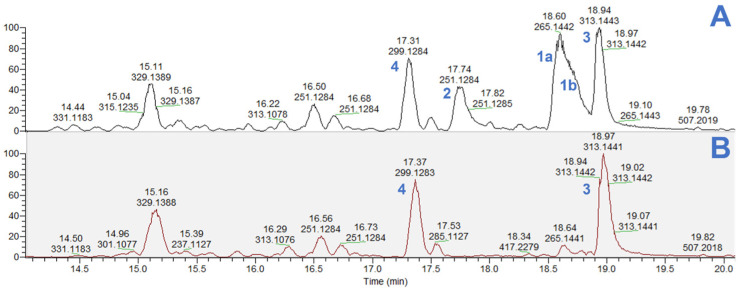
Negative mode base peak chromatogram of untreated (**A**) and steam-distilled (**B**) NZ Manuka hexane extract. Compound (**1**): a pair of isomers, leptospermone (**a**) and isoleptospermone (**b**); Compounds **2**–**4**: flavesone (**2**); grandiflorone (**3**); myrigalone A (**4**).

The mass spectrometry analysis of two NZ Manuka hexane extracts (Figure 2) also revealed that the untreated sample contained high concentrations of five β-triketones (Figure 2A), while only (**3**) and (**4**) could be detected in significant amounts in the steam-distilled sample (Figure 2B). The extraction of grandiflorone (**3**) and myrigalone A (**4**) through steam distillation is challenging due to the presence of benzene-containing substituents in their structure, which elevate their boiling points. Consequently, a significant quantity of these compounds persists in the leaves and branches after Manuka essential oil extraction.

Interestingly, in the *R_f_* 0–0.5 area of the TLC plate in Figure 1, steam-distilled Manuka contains significantly more antibacterial compounds than untreated Manuka. Due to the complex components, the present study has not conducted a detailed analysis of its chemical structure conversion. However, this result still demonstrated that heating may be an effective way to enhance the antibacterial ability of Manuka products.

### 2.2. Analysis of Flavonoids and Phloroglucinol Derivatives

**Figure 3:**
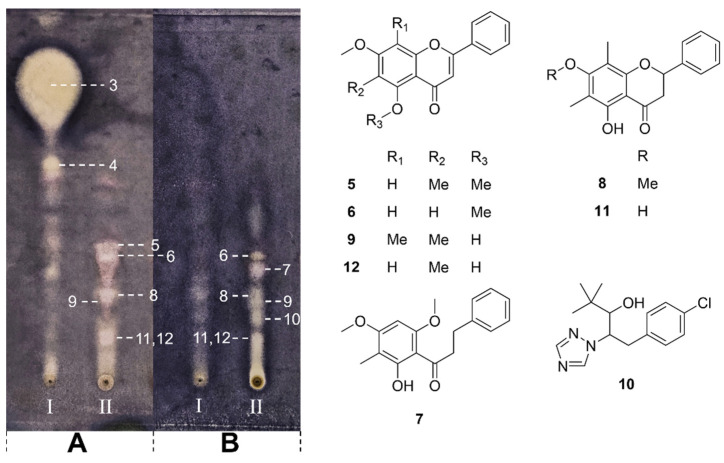
Bioautography assay of steam-distilled NZ Manuka ((**A**), 0.5 mg/mL, 3 μL spotting) and untreated CN Manuka ((**B**), 1 mg/mL, 3 × 3 μL spotting) extract against *S. aureus* at 37 °C, 24 h. Sample I: hexane extract; Sample II: untreated NZ Manuka. Compounds **3**–**12** are chemicals detected from the corresponding inhibition zones including β-triketones (**3**) and (**4**); phloroglucinol derivative (**7**); synthetic plant growth retardant, paclobutrazol (**10**); flavonoids (**5**, **6**, **8**, **9**, **11** and **12**).

We noticed that when the sample spotting amounts were the same (1 mg/mL, 3 μL spotting), the content of antibacterial ingredients in CN Manuka was almost unobservable, while the inhibition zones displayed by the two NZ Manuka extracts were significantly greater than CN sample and showed obvious overlap. In the LC-MS analysis, we found that the dichloromethane-extracted compounds of two Manuka samples were almost identical. In this section, we selected the steam-distilled NZ Manuka samples (0.5 mg/mL, 3 μL spotting) to compare with the CN Manuka samples (1 mg/mL, 3 × 3 μL spotting). The spotting volume was adjusted to allow all samples to present clearer inhibition zones, as shown in Figure 3. Despite this, we still did not isolate more active compounds from the hexane extract of NZ Manuka (Figure 3A-I), and like previous analysis, no clear inhibition zone can be observed from the hexane extract of CN Manuka (Figure 3B-I).

Six antibacterial flavonoids were identified from the dichloromethane extract of NZ Manuka (Figure 3A-II). The antibacterial compounds isolated from the dichloromethane extract of CN Manuka (Figure 3B-II) were largely the same as those in NZ Manuka and include two additional active compounds (**7**) and (**10**) that are different from NZ Manuka. The structures of isolated compounds and their positions on TLC were displayed in Figure 3. 

**Figure 4:**
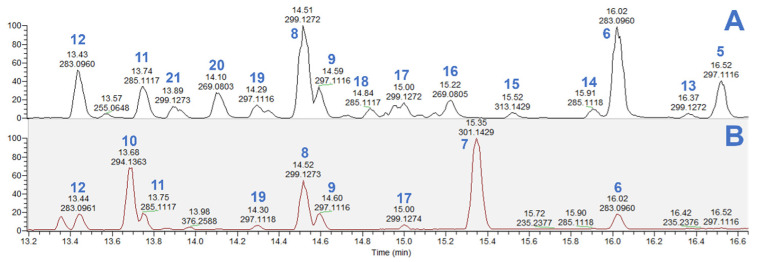
Positive mode base peak chromatogram of NZ (**A**) and CN (**B**) Manuka dichloromethane extract. Compounds **5**–**12**: chemicals detected from the corresponding inhibition zones shown in Figure 3. Compounds **13**–**21**: flavonoids and phloroglucinol derivatives identified that have not been collected from bioautography assay, structures shown in Figure 5.

The LC-MS analysis revealed that the NZ Manuka (Figure 4A) contains significantly more types of flavonoids compared with CN Manuka (Figure 4B). Compound (**7**) is a phloroglucinol derivative and was only presented in CN Manuka. However, compound (**10**) was identified as paclobutrazol, a synthetic chemical used as a plant growth retardant and fungicide [18]. The CN Manuka used in this study was cultured as an ornamental plant in China, so the discovery of paclobutrazol was not unexpected. It is worth noting that this study did not find β-triketones, the characteristic components of Manuka, from CN Manuka. The flavonoid content was also much less than NZ Manuka. This result is probably caused by the use of paclobutrazol. Paclobutrazol has been observed to elicit enzymatic and non-enzymatic antioxidant activities in plants [19]. One of its reported mechanisms of action involves interference with gibberellin biosynthesis by impeding the oxidation process from ent-kaurene to ent-kauronoic acid [20,21]. Based on this observation, we hypothesize that the mechanism of action of paclobutrazol also hampers the oxidation of phloroglucinol and phenylpropanoid in CN Manuka, subsequently inhibiting the biosynthesis of triketones [22] and flavonoids [23].

**Figure 5:**
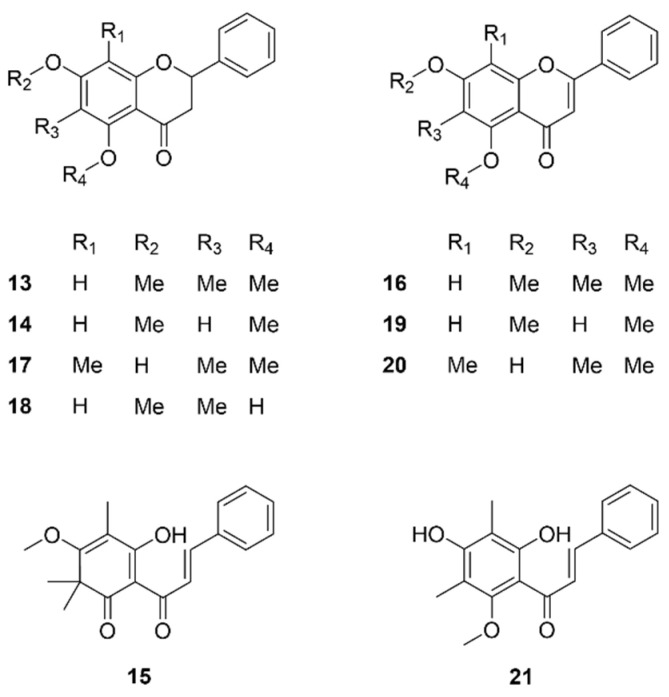
Chemical structures of low-content or inactive flavonoids (**13**,**14**, **16**–**20**) and phloroglucinol derivatives (**15** and **21**) identified from NZ Manuka dichloromethane extract.

The other flavonoids and phloroglucinol derivatives (**13**–**21**) with low content or which were not collected from the inhibition zone are identified and displayed in Figure 5.

### 2.3. Major Active Compound Purification and NMR Verification

The bioautography assay (above) and the matching of fragmentation patterns have determined that grandiflorone (**3**) was the primary antibacterial compound present in steam-distilled Manuka. Consequently, this section aims to validate the identification results and investigate the potential for the rapid purification of grandiflorone by employing the most straightforward and expeditious method, followed by structural verification using NMR.

**Figure 6:**
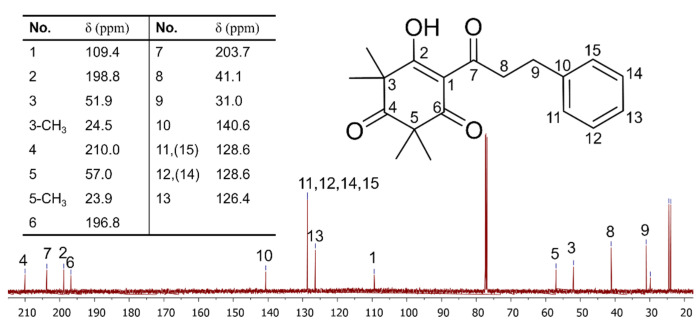
^13^C NMR spectrum (1D, 125 MHz, CDCl_3_) of grandiflorone isolated from steam-distilled NZ Manuka leaves and branches.

Two steps of normal-phase silica gel column chromatography yielded around 30 mg of grandiflorone (**3**) from steam-distilled NZ Manuka and carried an NMR analysis on the obtained product; the ^13^C spectrum is shown in Figure 6, and the full ^1^H, ^13^C and DEPT spectra are given in the supplementary data. Chemical shifts are given in δ (ppm), and compound **3** was confirmed to be grandiflorone. All chemical shifts matched previously reported data [17]. No obvious impurity signal can be observed in both the present spectra and HPLC detection, indicating that the target compound has been successfully purified from other phytochemicals.

The identical purification procedures were applied to untreated Manuka as well; however, the purity of the resulting grandiflorone was significantly lower compared to the utilization of steam-distilled Manuka. This outcome suggests that the process of extracting essential oil played a crucial role in concentrating the non-volatile β-triketones in plant materials, such as grandiflorone. Steam distillation rendered the by-product of Manuka essential oil extraction a valuable source for extracting grandiflorone.

### 2.4. Minimum Effective Dose of Grandiflorone

**Figure 7:**
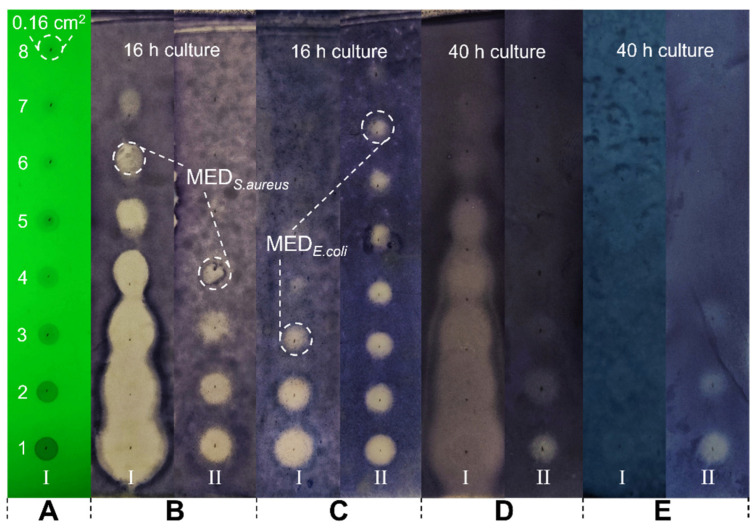
Direct bioautography-based minimum effective dose (MED) determination. Spots 1–8 on each plate were two-fold diluted samples with constant 3 μL spotting to control an approximately 0.16 cm^2^ sample area. Visualized under 254 nm UV light (**A**), inhibition effect against *S. aureus* ((**B**), 37 °C, 16 h; (**D**), 37 °C, 40 h) and *E. coli* ((**C**), 37 °C, 16 h; (**E**), 37 °C, 40 h). Sample I: grandiflorone, initial concentration 1 mg/mL; Sample II: tetracycline, initial concentration 0.1 mg/mL.

Different from conventional MIC testing, the minimum effective dose (MED) was defined in this study by the lowest amount of the tested sample to maintain an inhibition zone on the plate surface after 16–20 h. As shown in Figure 7, after eight consecutive two-fold dilutions of the grandiflorone (1 mg/mL), the solution of each concentration was evenly spotted on the TLC plate (Figure 7, Sample I). The adsorption area of the sample on the TLC plate was fixed at approximately 0.16 cm^2^ (example labeled in Figure 7A) by controlling the spotting volume at a constant 3 μL in one shot. This was followed by the conventional direct bioautography described in Section 3.5. Tetracycline (0.1 mg/mL, Figure 7, Sample II) was used as positive control and was subjected to the same treatment as grandiflorone.

The inhibition effect of grandiflorone against *S. aureus* (Figure 7A-I) and *E. coli* (Figure 7B-I) at 37 °C after 16 h can be directly observed from the plates. The spotted solution concentrations corresponding to the last clear inhibition zone are 31.25 μg/mL to the *S. aureus* and 250 μg/mL to the *E. coli*, respectively. According to the spotted volume and adsorption area of the chemicals, the MED of grandiflorone can be calculated as 0.29–0.59 μg/cm^2^ against *S. aureus* and 2.34–4.68 μg/cm^2^ against *E. coli*.

As a comparison, the minimum inhibitory concentration (MIC) of isolated grandiflorone was measured by the conventional micro broth dilution method; the MIC value against *S. aureus* and *E. coli* was 15.63–31.25 μg/mL and 250–500 μg/mL, respectively, as expected from previous reports [7,12]. The results of the two experiments were close, and grandiflorone showed a potent inhibition effect against *S. aureus* while requiring about 10 times the dose against *E. coli.* From our existing data, TLC-MED determination requires much less of the sample than conventional MIC testing, and the results were clear. We found that it is very suitable for non-water-soluble compound testing and solved the interference caused by certain compounds having a strong color in liquid, which can interfere with the absorbance reading.

The sustained antibacterial effect of grandiflorone was also recorded. Figure 7D,E exhibited the inhibition zones after 40 h of incubation; even at the highest dose, the effect against *E. coli* was completely disappeared (Figure 7E-I), while the effect against *S. aureus* just started dissipating (Figure 7D-I) until the TLC plate dried out (greater than 48 h). It can be inferred that bacteria will progressively occupy the inhibition zones over a certain period. Consequently, in forthcoming research, we intend to investigate the time necessary for the inhibition zone to be colonized by bacteria at a specific molar dosage. This parameter will serve as an evaluative measure of the compound’s susceptibility.

The conventional direct bioautography method was found to be unsuitable for osmotically vulnerable bacteria, such as *E. coli*, due to the drying out of TLC plates during incubation, resulting in a high osmotic pressure environment. This limitation necessitates the use of agar overlay bioautography in numerous studies [24,25,26]. Although one direct TLC-bioautography protocol exists for optimizing *E. coli* growth conditions, the 5 h incubation time was insufficient for evaluating the antibacterial activity of compounds [27]. To ensure *E. coli* survival, we replaced Petri dishes with aluminum foil-wrapped glass containers and added moisture through spraying and water-saturated absorbent cotton. Using this approach, we effectively ascertained the viability of *E. coli* on the TLC surface for a duration exceeding 48 h. Throughout this timeframe, the addition of water to the absorbent cotton could be employed to extend the survival of *E. coli*. Although we initially attempted to encase the container with plastic wrap, we observed that water would drop onto the TLC surface during the culturing procedure, thereby significantly compromising the ultimate experimental results. Consequently, aluminum foil presently stands as the optimal wrapping material.

Like the disk diffusion method, TLC-MED determination is also an observation of the inhibition zone, but we do not think that the diffusion diameter of the zones has a strong correlation with the antibacterial ability of the compound tested. Both experiments share a common shortcoming: the size of the zones is also affected by the diffusion ability of the sample on the attached matrix. As shown in Figure 7B,C, either against *S. aureus* or *E. coli*, there is a significant difference in the diameter of the inhibition zone of sample I (grandiflorone) from high dose to low dose. However, the difference in the diameter of the inhibition zone presented by sample II (tetracycline) within the effective dose range was not obvious. This finding contradicts the observations made in previous experiments conducted with the disk diffusion method. In the case of conventional water-based semisolid media, the non-polar compound grandiflorone exhibited insolubility, impeding its diffusion, whereas tetracycline demonstrated efficient diffusion. Conversely, on the TLC plate, the silica gel became saturated with the liquid culture medium, facilitating the desorption and diffusion of non-polar compounds. Therefore, the size of the inhibition zone was not employed as a parameter to assess antibacterial efficacy in this study.

## 3. Materials and Methods

### 3.1. Chemicals and Reagents

Methanol, n-hexane, dichloromethane, and acetone for extraction and chromatography were analytical research-grade; acetonitrile, methanol, and formic acid used for instrumental analysis were LC-MS-grade (Merck, Darmstadt, Germany). Chloroform-d (99.9%, Sigma-Aldrich, St. Louis, MO, USA) was used for nuclear magnetic resonance (NMR). Normal phase thin-layer chromatography (TLC, silica gel F_254_, 0.2 mm in thickness, aluminum sheets), silica gel for column chromatography (40 μm, Sigma-Aldrich, USA). 3-(4,5-Dimethylthiazol-2-yl)-2,5-Diphenyltetrazolium Bromide (MTT, 99%, AK Scientific, Union City, CA, USA) stock solution, 5 mg/mL, and tetracycline (98%, Sigma-Aldrich, USA) stock solution, 0.1 mg/mL, were prepared in sterile water and stored at 4 °C in the dark.

### 3.2. Plant Material

*Leptospermum scoparium* samples were collected from New Zealand and China, respectively. New Zealand (NZ) Manuka was harvested from the East Cape area of New Zealand provided by Manuka Biologicals company, including untreated and steam-distilled samples (essential oil distillation by-products). Chinese (CN) Manuka was purchased from China, Fujian province, Longyan city, and was untreated. The sample was further confirmed to be *Leptospermum scoparium* by Jennifer Tate, Massey University. The specimen (storage code: MOMP-1) of NZ Manuka and extract of CN Manuka (storage code: MSHM-1) were stored at the Massey University’s Manawatū campus, Science Tower cold storage room. The present study focused on the leaves and branches. Manuka samples were air-dried and ground into a fine powder (60–80 mesh) using a grinding mill (HC-2000Y, Wuyi Haina Electric Appliance Co., Ltd., Jinhua, China). The dry weight of each sample was 200 g.

### 3.3. Extraction of Phytochemicals

The powders of three Manuka samples (untreated NZ Manuka, steam-distilled NZ Manuka, and untreated CN Manuka) were extracted using 1000 mL of methanol in a Soxhlet extractor for 12 h, respectively. The temperature in the thimble during extraction was approximately 60 °C. Methanol extracts obtained from each sample were concentrated on a rotary evaporator at 35 °C to yield gum-like crude extracts. Then, the crude extracts were suspended in water, and liquid–liquid extracted by hexane and dichloromethane, respectively, to separate compounds based on polarity.

### 3.4. Bacteria Cultivation and Conventional Antibacterial Assay

Gram-positive *Staphylococcus aureus* (ATCC 25923) and Gram-negative *Escherichia coli* (ATCC 25922) strains were used in this study. The strains were obtained from the Microbiology Department, Massey University, and stored in brain–heart infusion (BHI, Difco) agar slants at 4 °C. For each experiment, a single colony was freshly picked and cultured in BHI broth at 37 °C for 24 h.

The broth microdilution method was employed to assess the minimum inhibition concentration (MIC) of purified compounds. The assay was performed on a 96-well plate, bacterial suspensions were 100 times diluted after overnight culture, tested samples were 2-fold diluted from 500 μg/mL, and tetracycline (100 μg/mL) was used as positive control. The lowest concentration that remained clear after overnight incubation was determined as the MIC.

### 3.5. TLC Analysis and Direct Bioautography

#### 3.5.1. TLC Analysis

The hexane and dichloromethane extracts were dissolved at a concentration of 1 mg/mL for TLC analysis. A mixture of n-hexane and acetone (7:3, *v*:*v*) was used as the developing agent. The analysis was performed in a rectangular TLC developing chamber. The chamber was shaken with the developing agent and sealed with parafilm while developing. The plates were removed when the solvent front had moved to 90% of the total plate length (10 cm) and subsequently allowed to dry. The results were visualized under 254 nm UV light (Dark Box UV lamp, Qiwei ZF-1, Hangzhou Qiwei Instrument Co., Ltd., Hangzhou, China) followed by UV sterilization and bioautography experiments.

#### 3.5.2. Direct TLC Bioautography

Direct bioautography was performed by the procedure described in these articles [28,29]. A bacterial suspension with an optical density of 1.2 was newly prepared before each test. TLC plates with separated compounds were dried in a fume hood overnight, sterilized with UV light for 20 min and immersed in the bacterial suspension for 10 s and stored in a glass container, wrapped with aluminum foil. For TLC with *S. aureus*, the wrapped containers were directly incubated at 37 °C for 16 h. However, this process was not suitable for osmotically vulnerable bacteria, such as *E. coli*. The present study applied an improved direct bioautography protocol. That is, for TLC with *E. coli*, the inner walls of the container (1 L) were sprayed with a layer of water mist, and water-saturated absorbent cotton (10 mL) was placed in the corner of the container before wrapping, then incubated at 37 °C for 16 h in total. We discovered that this treatment method could best ensure the effective duration of the experiment and the best survival of bacteria. For visualization of microbial growth, MTT solution was sprayed onto the plates and re-incubated at 37 °C for 3 h. Clear white zones against a bluish-violet background on the TLC plate indicate the anti-microbial activity of the compounds [29,30,31]; the white zone is referred to as the inhibition zone in this study.

#### 3.5.3. Direct Bioautography-Based Minimum Effective Dose Determination

This experiment determined the minimum effective dose (MED) of tested chemicals by continuously reducing the amount of sample spotted. The minimum dose that maintained a clear inhibition zone on the plate after 16–20 h was defined as the MED. To ensure that the sample area on the TLC plate was consistent, the spotting volume throughout the study was unified to 3 μL, and a pipette was used to complete the spotting. The tested sample solutions were first diluted into several concentration gradients, then cultured and stained as described in the above-mentioned procedures after spotting.

### 3.6. Rapid Identification of Antibacterial Compounds

The main compound(s) related to an inhibition zone were selected as the target compounds in this study. For rapid identification, each target compound to be analyzed was located by retention factor (*R_f_*) value and was collected by scraping the silica gel from other newly developed TLC plates without bacteria and flushed with acetone. The hexane and dichloromethane extracts (1 mg/mL) and each collected target compound were analyzed using UHPLC-HR-MS (Thermofisher Dionex Ultimate 3000 High-Performance Liquid Chromatography and Q Executive Focus High-Resolution Mass Spectrometer) and were preliminary identified through online database-matching performed using Compound Discoverer 3.3. The datasets used in the matching process include mzClound, ChemSpider, and Mass Lists. The LC-MS raw data, workflow employed, Mass Lists details, and the identification reports generated using Compound Discoverer were provided in the supplementary data. The database matching cannot appropriately distinguish the flavonoid isomers; therefore, the structures obtained were further revised with reference to the reported chemical composition of Manuka accomplished by other researchers [32,33,34,35]. The separation was performed using a Thermofisher Hypersil GOLD C18 Column, 100 × 2.1 mm, particle size 1.9 μm. Solvent A water/0.1% formic acid, solvent B acetonitrile/0.1% formic acid. Running method for hexane extracts: 0–4 min 5–45% B, 4–8 min 45% B, 8–16 min 45–95% B, 16-25 min 95% B, 25–26 min 95–5% B, 26–30 min 5% B, 0.2 mL/min flow rate. Running method for dichloromethane extracts: 0–3 min 5% B, 3–15 min 5–95% B, 15–18 min 95% B, 18–23 min 95–5% B, 0.3 mL/min flow rate. The inject volume was set to 5 μL, and column oven was maintained at 25 °C. Mass spectra were acquired under an electrospray ionization (ESI) source in both positive and negative ionization modes through full MS and data-dependent MS/MS analysis. The optimal MS parameters were set as follows: spray voltage, 2.5 kV; capillary temperature, 350 °C; sheath gas, 35 units; auxiliary gas, 10 units; auxiliary gas heater temperature, 200 °C; sweep gas, 5 units; collision energy, 30 eV. The mass range was set from 120 to 1500 *m*/*z* at a resolution of 70,000.

### 3.7. Major Active Compounds Purification

Normal phase silica gel column chromatography was used to isolate the major active compounds related to the maximal inhibition zone. The hexane extracts ~3 g were loaded on a manually packed column (4 cm diameter, 50 g silica gel), and the separation was conducted using a flash chromatography apparatus (BÜCHI, control unit C-620; Fraction Collector C-660; Pump Module C-605 and UV Photometer C-640) with eluent n-hexane (Solvent A) and dichloromethane (Solvent B). Separation method:0–5 min 0% B, 5–6 min 0–5% B, 6–25 min 5–15%, 25–30 min 15–30% B, 30 mL/min flow rate. The UV detector was set to 220 nm and 254 nm, collector was set to collect fractions (peaks) detected by 254 nm, 10 mL/tube. Each tube was subjected to TLC examination to target compounds. Semi-purified compounds ~100 mg were recovered through reduced pressure distillation and subjected to another column (1 cm diameter, 10 g silica gel) with the same eluent solvents and the same collection and detection settings. Separation method:0–10 min 5% B, 10–20 min 5–6% B, 20–25 min 6–7%, 25–30 min 7–30% B, 5 mL/min flow rate. The final yield of the pure compound was ~30 mg. The purity of the isolated compound was examined using HPLC using the area normalization method on triplicate experiments; the detection was completed with a DAD (diode array detector, Dionex, Ultimate 3000) at 220 nm and 254 nm, respectively, performed in the same column described in Section 3.6. Running method: linear increases acetonitrile from 45% to 95% in water, 10 min, 5 μL inject volume, 0.2 mL/min flow rate at 25 °C.

### 3.8. Nuclear Magnetic Resonance (NMR) 

We verified the structure of major active compounds identified using Compound Discoverer as well as for detecting the possible existing non-UV-active organic compounds (impurities) derived from the extract. The ^1^H, ^13^C and Distortionless Enhancement by Polarization Transfer (DEPT 135°) NMR (Bruker AV 500 spectrometer) were used. The analyte was prepared in chloroform-d. The chemical shifts were reported in parts per million (ppm) downfield from Tetramethylsilane (TMS). The NMR raw spectrums were provided in the Supplementary data.

### 3.9. Data Acquisition and Software Analysis

Images of TLC plates were processed via ImageJ (National Institutes of Health, New York, NY, USA, version 1.53t). The chemical structure diagrams were edited using ChemBioDraw (CambridgeSoft, Cambridge, MA, USA, version 14.0). The Mass data acquisition and analysis were performed by Xcalibur (Thermofisher, Waltham, MA, USA, version 4.4). The compound identification was performed using Compound Discoverer (Thermofisher, USA, version 3.3) through ChemSpider, mzCloud, mzVault and Mass List library matching. The NMR spectrum was analyzed using MestReNova (Mestrelab Research, Santiago de Compostela, Spain, version 6.1.0). 

## 4. Conclusions

The present study rapidly targeted and identified the antibacterial compounds presented in untreated, steam-distilled NZ Manuka and untreated CN Manuka, respectively. A total of **22** compounds were identified through LC-MS analysis and by matching with the Compound Discoverer library, including β-triketones (**1a**, **1b** and **2**–**4**), flavonoids (**5**, **6**, **8**, **9**, **11**–**14** and **16**–**20**), phloroglucinol derivatives (**7**, **15** and **21**), and a synthetical plant growth retardant (**10**). Compounds **1**–**12** were the major antibacterial compounds isolated through direct TLC-bioautography-guided separation. Among them, five β-triketones were only detectable in two NZ Manuka samples, compounds (**7** and **10**), which were only present in CN Manuka; the types of flavonoids detected in CN Manuka were also much less than those in NZ Manuka. The reason for this phenomenon may be that a large amount of growth retardant (**10**) was applied to CN Manuka which was cultured as an ornamental plant. Compounds **13**–**21** were low-content or inactive flavonoids and phloroglucinol derivatives identified through LC-MS analysis directly. 

The comparative TLC and LC-MS analysis between untreated and steam-distilled NZ Manuka revealed that a large amount of grandiflorone (**3**) and myrigalone A (**4**) remained in the leaves and branches after Manuka essential oil extraction, due to their low volatility. The column chromatography was conducted to purify grandiflorone (**3**) identified through LC-MS analysis from both untreated and steam-distilled NZ Manuka. Subsequently, NMR analysis confirmed that compound **3** was indeed grandiflorone. Notably, the results of this purification process demonstrated that steam-distillation treatment significantly alleviated the challenges associated with purifying grandiflorone. Consequently, Manuka leaves and branches, which are retained after essential oil production, emerged as a more favorable source for extracting grandiflorone compared to fresh Manuka.

We also described an improved TLC-bioautography protocol for assessing the antibacterial ability of grandiflorone. The minimum effective dose (MED) was observed to be 0.29–0.59 μg/cm^2^ against *S. aureus* and 2.34–4.68 μg/cm^2^ against *E. coli*. The enhanced protocol demonstrated suitability for both *S. aureus* and *E. coli.* The obtained results were unambiguous and intuitive, and remain unaffected by the solubility, diffusion capacity, or color of the test compounds, in contrast to the conventional antibacterial assay.

## Data Availability

All raw data and experimental details are available upon request.

## Supplementary Materials

The following supporting information can be downloaded at: https://www.mdpi.com/article/10.3390/molecules29030717/s1, Supplementary data A. Workflow used in Compound Discoverer 3.3. Supplementary data B. Databases included in MassList. Supplementary data C. LC-MS raw data of Manuka hexane and dichloromethane extracts. Supplementary data D. ^1^H, ^13^C and DEPT NMR spectrum of obtained Grandiflorone. Supplementary data E. Compound identification report generated by Compound Discoverer.
