# Supplementary material for: TLC-Bioautography-Guided Isolation and Assessment of Antibacterial Compounds from Manuka (Leptospermum scoparium) Leaf and Branch Extracts
Source: Molecules. 2024 Feb 4;29(3):717. doi: 10.3390/molecules29030717 (PMC10856334; doi:10.3390/molecules29030717)
Supplement: Supplementary file 1 [file molecules-29-00717-s001.zip › molecules-2827338-supplementary.pdf]

Supplementary data

Supplementary data A. Workflow used in Compound Discoverer 3.3.

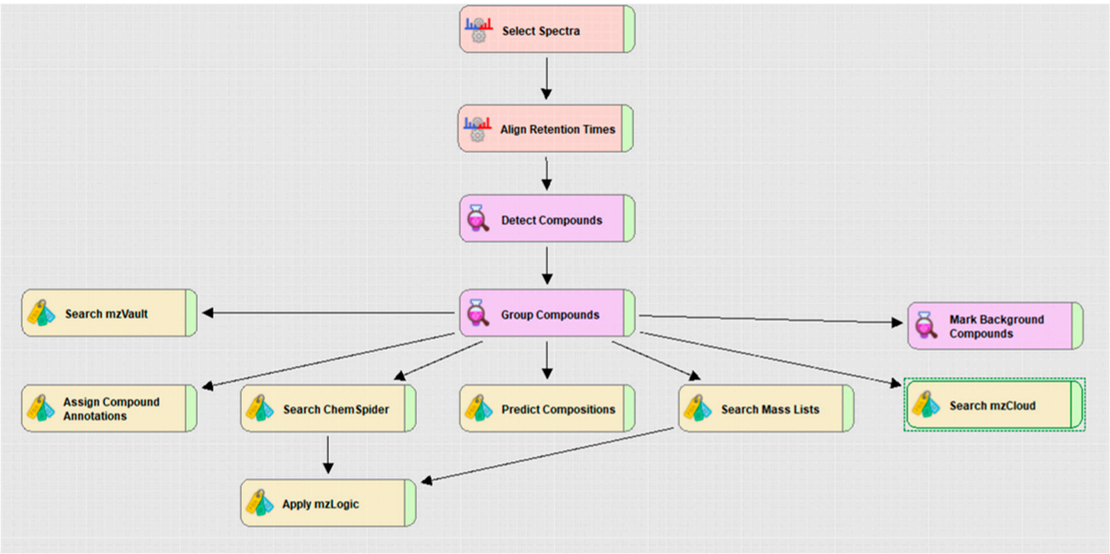

Supplementary data B. Databases included in MassList:

1. Arita Lab 6549 Flavonoid Structure Database
2. EFS HRAM Compound Database
3. Endogenous Metabolites Database
4. Example Mass List
5. Extractables and Leachables HRAM Compound Database
6. Lipid Maps Structure Database
7. Natural Products Atlas 1
8. Natural Products Atlas 2

Supplementary data C. LC-MS raw data of Manuka hexane and dichloromethane extracts.

C-1: LC-MS analysis of Manuka hexane extracts. (1) NZ untreated; (2) NZ steam-distilled; (3) CN untreated

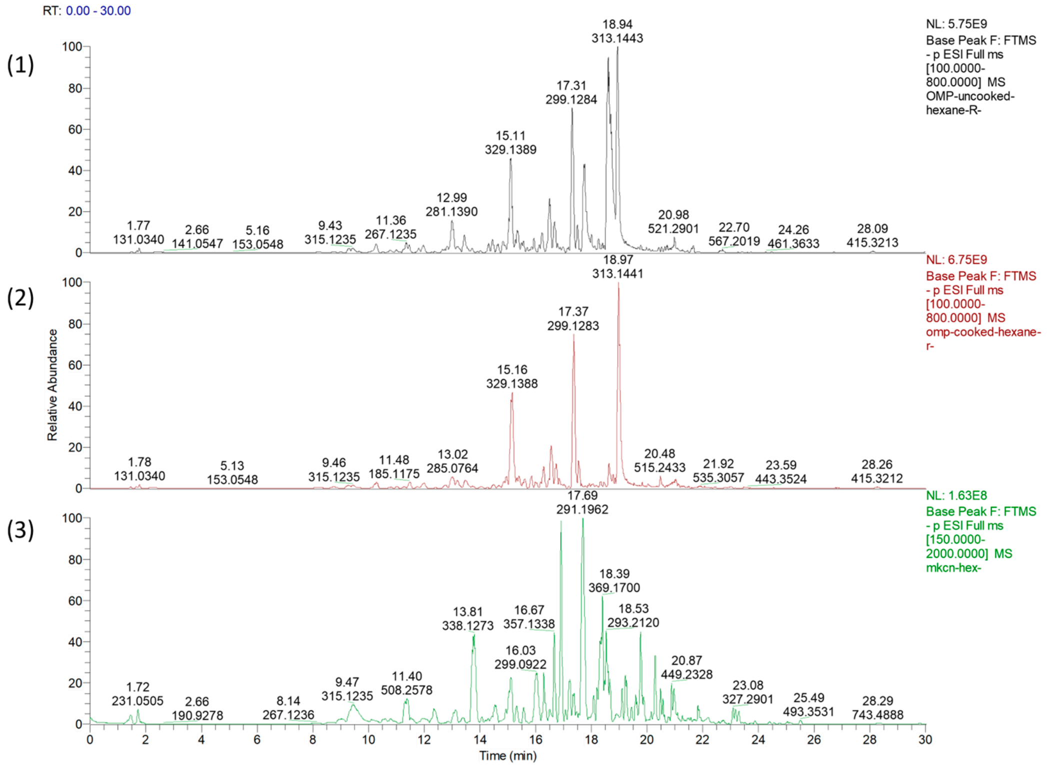

C-2: LC-MS analysis of Manuka dichloromethane extracts. (1) NZ untreated (2) CN untreated

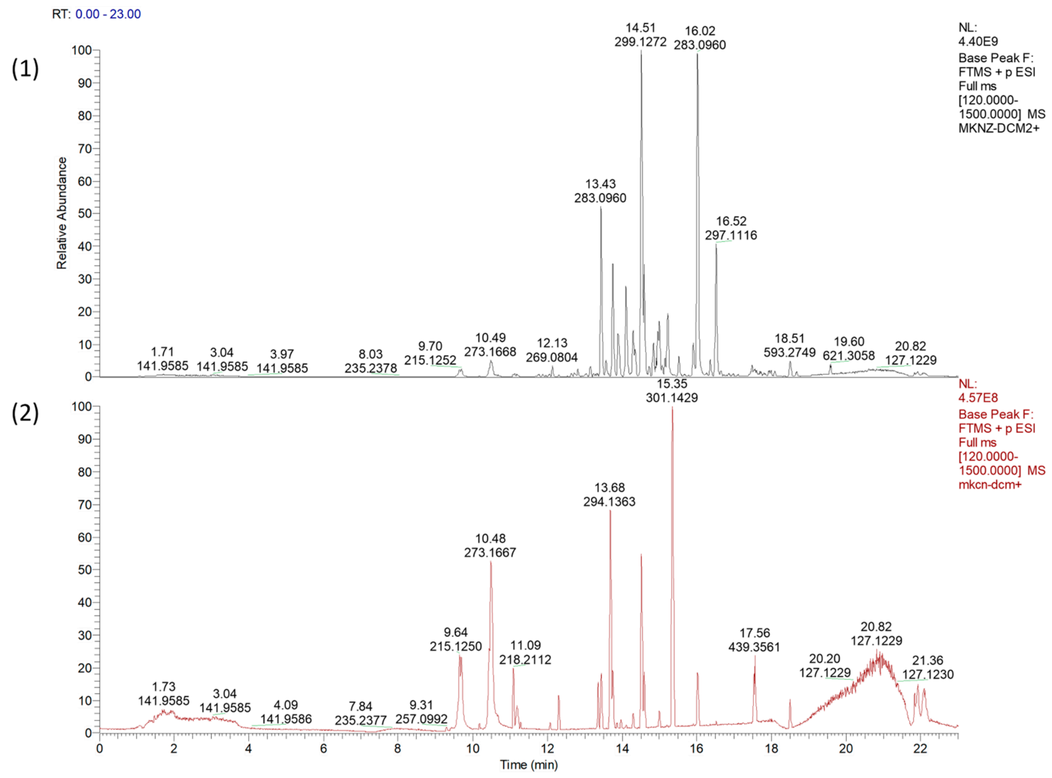

Supplementary data D. ^{1}H,^{13}C and DEPT NMR spectrum of obtained Flavone.

^{1}H NMR original graph:

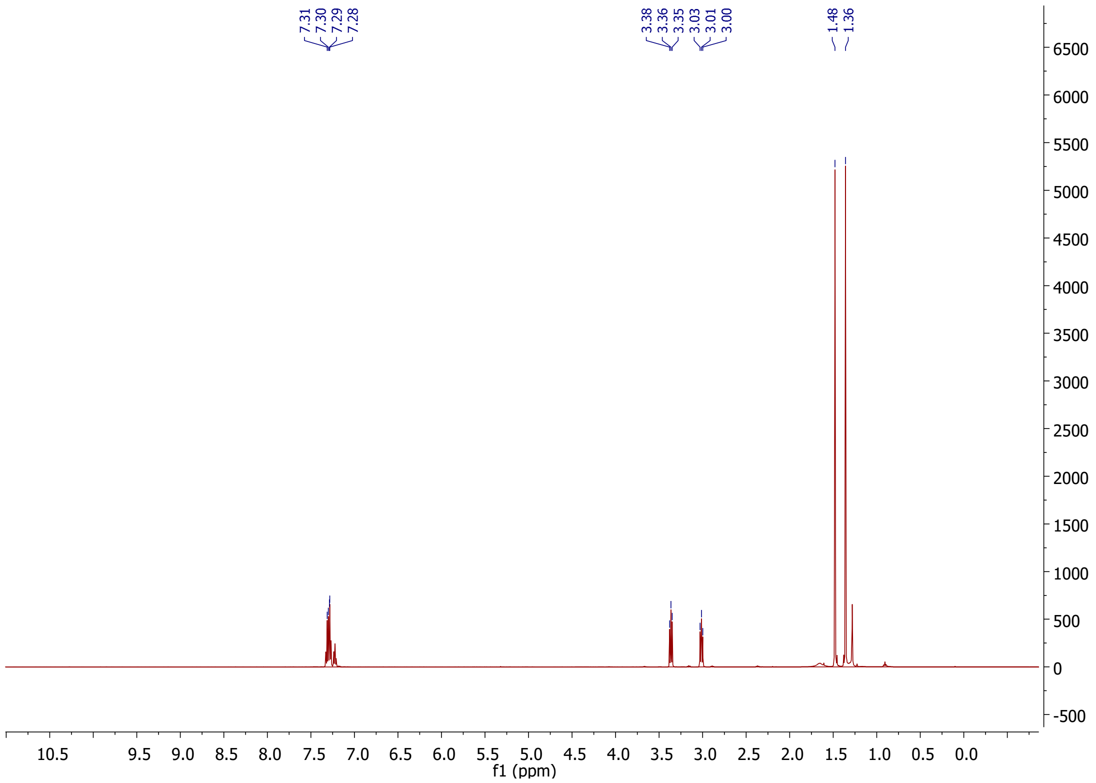

^{1}H NMR partial magnified graph:

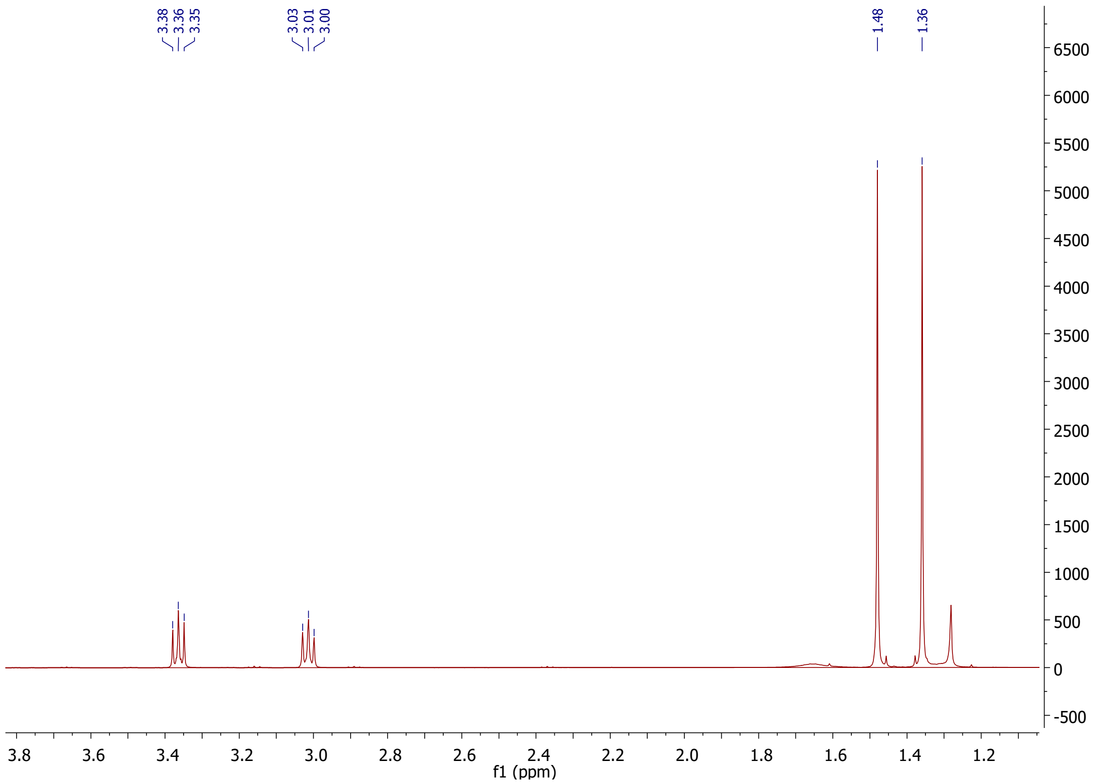

^{13}C NMR original graph:

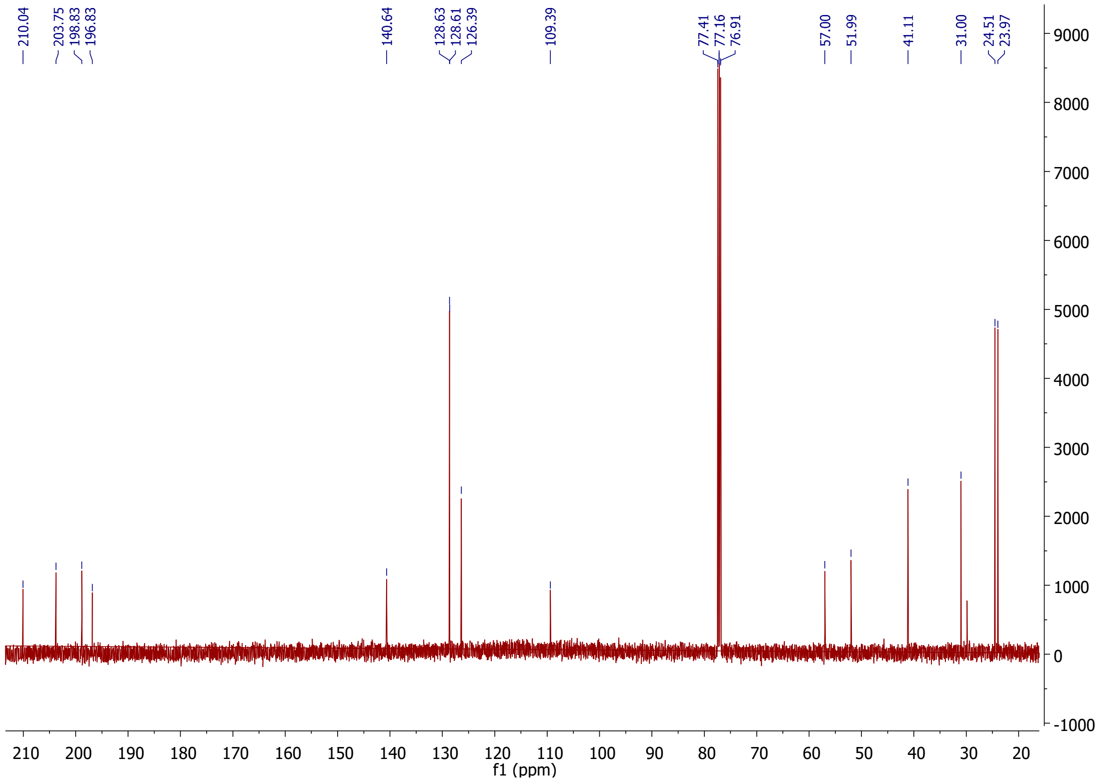

DEPT 135° NMR original graph:

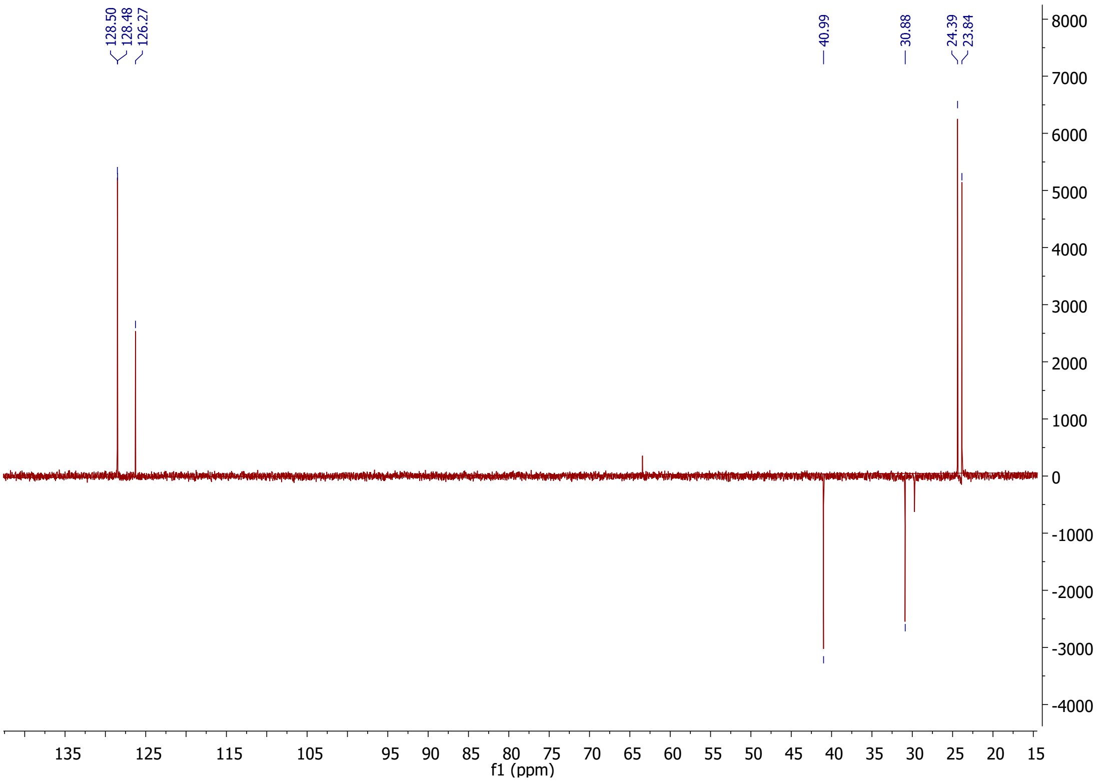

Supplementary data E. Compound identification report generated by Compound Discoverer.

E-1: mzCloud matching results.

Name	Formula	Calc. MW	m/z	RT [min]	Area (Max.)	# ChemSpider Results	mzCloud Best Match	Mass List Matches	mzVault Best	mzVault Library	MS2	Reference Ion	Area	Peak Rating
5,7-Dimethoxy-6-methyl-2-phenyl-4H-chromen-4-one	C18 H16 O4	296.10430	297.11157	16.519	5977294677	84	78.6					[M+H]+1	5.98e9	9.5
5-Hydroxy-7-methoxy-6-methylflavon	C17 H14 O4	250.06249	283.09598	16.020	15327890311	19	95.0					[M+H+MeOH +1	1.53e10	9.5
Tectochrysin	C16 H12 O4	236.04698	269.08047	15.222	3340663941	5	96.7		53.0			[M+H+MeOH +1	3.34e9	9.5
5,7-Dimethoxy-6-methyl-2-phenyl-4H-chromen-4-one	C18 H16 O4	296.10430	297.11160	14.292	1942783765	84	61.7					[M+H]+1	1.94e9	9.5
5,7-Dihydroxy-6-methyl-2-phenyl-4H-chromen-4-one	C16 H12 O4	268.07308	269.08035	14.102	4426294416	111	99.1					[M+H]+1	4.43e9	8.9
5-Hydroxy-7-methoxy-6-methylflavon	C17 H14 O4	282.08872	283.09601	13.436	6788206946	118	72.9					[M+H]+1	6.79e9	10.0
Paclobutrazol	C15 H20 Cl N3 O	293.12889	294.13617	13.686	921313130	24	99.9		95.6			[M+H]+1	9.21e8	9.5

E-2: Mass Lists matching results.

Structure	Name	RT [min]	Formula	Calc. MW	m/z	Mass List Matches
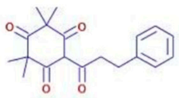	2,2,4,4-Tetramethyl-6-(3-phenylpropanoyl)-1,3,5-cyclohexanetrione	18.94	C19 H22 O4	314.15159	313.14432	

Structure	Name	RT [min]	Formula	Calc. MW	m/z	Mass List Matches
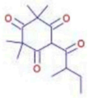	Isoleptospermone	18.60	C15 H22 O4	266.15144	265.14417	

Structure	Name	RT [min]	Formula	Calc. MW	m/z	Mass List Matches
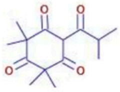	Flavesone	17.76	C14 H20 O4	252.13569	251.12842	

Structure	Name	RT [min]	Formula	Calc. MW	m/z	Mass List Matches
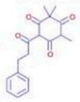	2,2,4-Trimethyl-6-(3-phenylpropanoyl)-1,3,5-cyclohexanetrione	17.31	C18 H20 O4	300.13573	299.12845	

Structure	Name	RT [min]	Formula	Calc. MW	m/z	Mass List Matches
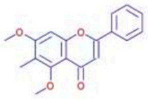	5,7-Dimethoxy-6-methyl-2-phenyl-4H-chromen-4-one	16.52	C18 H16 O4	296.10430	297.11157	

Structure	Name	RT [min]	Formula	Calc. MW	m/z	Mass List Matches
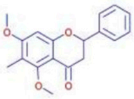	5,7-Dimethoxy-6-methyl-2-phenyl-2,3-dihydro-4H-chromen-4-one	16.36	C18 H18 O4	298.11995	299.12723	

Structure	Name	RT [min]	Formula	Calc. MW	m/z	Mass List Matches
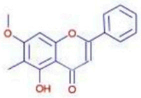	5-Hydroxy-7-methoxy-6-methylflavon	16.02	C17 H14 O4	250.06249	283.09598	

Structure	Name	RT [min]	Formula	Calc. MW	m/z	Mass List Matches
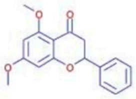	5,7-Methoxyflavanone	15.91	C17 H16 O4	284.10448	285.11176	

Structure	Name	RT [min]	Formula	Calc. MW	m/z	Mass List Matches
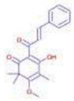	3-Hydroxy-5-methoxy-4,6,6-trimethyl-2-[(2E)-3-phenyl-2-propenoyl]-2,4-cyclohexadien-1-one	15.52	C19 H20 O4	312.13566	313.14294	
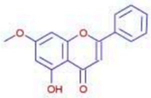	Tectochrysin	15.22	C16 H12 O4	236.04698	269.08047	
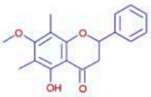	5-Hydroxy-7-methoxy-6,8-dimethylflavanone	15.00	C18 H18 O4	298.11987	299.12717	
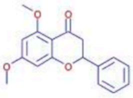	5,7-Methoxyflavanone	14.84	C17 H16 O4	284.10442	285.11169	
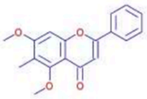	5,7-Dimethoxy-6-methyl-2-phenyl-4H-chromen-4-one	14.59	C18 H16 O4	296.10427	297.11157	
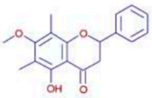	5-Hydroxy-7-methoxy-6,8-dimethylflavanone	14.52	C18 H18 O4	298.11988	299.12717	
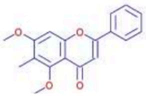	5,7-Dimethoxy-6-methyl-2-phenyl-4H-chromen-4-one	14.29	C18 H16 O4	296.10430	297.11160	
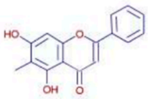	5,7-Dihydroxy-6-methyl-2-phenyl-4H-chromen-4-one	14.10	C16 H12 O4	268.07308	269.08035	

Structure	Name	RT [min]	Formula	Calc. MW	m/z	Mass List Matches
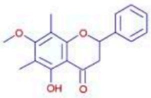	5-Hydroxy-7-methoxy-6,8-dimethylflavanone	13.89	C18 H18 O4	298.11999	299.12729	
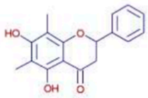	Demethoxymatteucionol	13.75	C17 H16 O4	284.10434	285.11160	
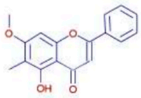	5-Hydroxy-7-methoxy-6-methylflavon	13.44	C17 H14 O4	282.08872	283.09601	
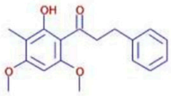	1-(2-Hydroxy-4,6-dimethoxy-3-methylphenyl)-3-phenyl-1-propanone	15.35	C18 H20 O4	150.06780	301.14288	
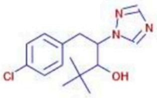	Paclobutrazol	13.69	C15 H20 Cl N3 O	293.12889	294.13617	
